# Differential Expression of *TFF1* and *TFF3* in Patients Suffering from Chronic Rhinosinusitis with Nasal Polyposis

**DOI:** 10.3390/ijms20215461

**Published:** 2019-11-01

**Authors:** Martina Mihalj, Maro Bujak, Josip Butković, Željko Zubčić, Maja Tolušić Levak, Josip Čes, Vlatko Kopić, Mirela Baus Lončar, Hrvoje Mihalj

**Affiliations:** 1 Department of Dermatology and Venereology, University Hospital Osijek, 31000 Osijek, Croatia; martina.mihalj@mefos.hr (M.M.); mtolusic@mefos.hr (M.T.L.); 2 Department of Physiology and Immunology, Faculty of Medicine, University of Osijek, 31000 Osijek, Croatia; 3 Department of Materials Chemistry, Ruđer Bošković Institute, 10000 Zagreb, Croatia; Maro.Bujak@irb.hr; 4 Department of Maxillofacial Surgery, University Hospital Osijek, 310000 Osijek, Croatia; butkovic.josip@kbo.hr (J.B.); kopic.vlatko@kbo.hr (V.K.); 5 Department of Otorhinolaryngology and Maxillofacial Surgery, Faculty of Medicine, University of Osijek, 31000 Osijek, Croatia; zubcic.zeljko@kbo.hr; 6 Department of Otorhinolaryngology and Head and Neck Surgery, University Hospital Osijek; 31000 Osijek, Croatia; 7 Department of Histology and Embryology, Faculty of Medicine, University of Osijek, 31000 Osijek, Croatia; 8 Dental Centre Čes, 31000 Osijek, Croatia; josip.ces@gmail.com; 9 Department of Molecular Medicine, Ruđer Bošković Institute, 10000 Zagreb, Croatia

**Keywords:** trefoil factor family, chronic rhinosinusitis, nasal polyposis

## Abstract

Trefoil family factor (TFF) proteins contribute to antimicrobial defense and the maintenance of sinonasal epithelial barrier integrity. Dysregulation of TFF expression may be involved in the development of chronic inflammation and tissue remodeling characteristically found in chronic rhinosinusitis with nasal polyposis (CRSwNP). Expressions of *TFF1* and *TFF3* were determined in specimens of middle nasal turbinate (MNT-0), bulla ethmoidalis (BE), and nasal polyps (NP) from CRSwNP patients (*n* = 29) and inferior nasal turbinate from a group of control patients (underwent nasal septoplasty, *n* = 25). An additional MNT sample was collected 6 months after functional endoscopic sinus surgery (FESS, MNT-6). *TFF1* mRNA levels were significantly reduced in all specimens by approximately three- to five-fold, while *TFF3* was increased in MNT-0, as compared with controls. Six months after surgery their levels were reversed to control values. CRSwNP patients with *S. epidermidis* isolated from sinus swabs showed upregulation of *TFF3* in MNT and NP as compared with patients with sterile swabs. Target gene regulation was not affected by the presence of type 2 inflammation in patients with confirmed allergy. Results of this study imply participation of *TFFs* genes in the development of CRSwNP.

## 1. Introduction

Chronic rhinosinusitis (CRS) is defined as an inflammatory condition that is affecting the paranasal sinuses and nasal cavity persistently for more than 12 weeks [1]. Furthermore, it is divided into two different phenotypes, CRS without nasal polyposis (CRSsNP) and CRS with nasal polyposis (CRSwNP). There is no clear understanding of the underlying pathophysiology that causes chronic rhinosinusitis and the uncontrolled tissue proliferation leading to formation of mucosal outgrowths in the form of nasal polyps. However, several contributors have been implicated in the etiology of CRSwNP, including the presence of certain microorganisms, formation of biofilms, bacteria-derived superantigens, dysregulation of the innate and adaptive immunity, and, finally, the presence of asthma and intolerance to the inhibitors of the cyclooxygenase-1 enzyme which together with CRSwNP form aspirin-exacerbated respiratory disease (AERD) [2]. In his study, Pawankar described four different histological types of nasal polyps, with the eosinophilic type and chronic inflammatory type as the most common [3]. The other two nasal polyp types are rarely found among CRSwNP patients, and histologically they are characterized by pronounced hyperplasia of seromucinous glands or with atypical stroma [4].

Trefoil factor family (TFF) peptides, including TFF1, TFF2, and TFF3 are a small group of molecules that bear a characteristic three-loop trefoil domain and play a central role in the maintenance of epithelial surface integrity [5,6,7,8]. The presence of TFF peptides has been reported in various tissues including the brain, lungs, trachea, thyroid gland, salivary glands, prostate, uterus, liver, and most abundantly in the mucosa lining the gastrointestinal (GI) tract. In the airway, they have been identified within the mucosa of the upper and lower respiratory tract, mostly in goblet cells, ciliated cells, and in some submucosal cells [9]. Their expression was confirmed in the respiratory mucosa of healthy human subjects, as well as in patients suffering from inflammation-driven diseases, such as cystic fibrosis, chronic sinusitis, and bronchitis, however, in the latter case, with diverse expression pattern as compared with uninflamed mucosa [9,10]. In a study by Li and Turner, expression of *TFF1* and *TFF3* was significantly higher in mucosa from CRSsNP patients, while there was no difference between controls and CRSwNP patients [10]. A recent study reported increased *TFF1* gene and protein expression in patients suffering from either CRSsNP or nasal polyposis without CRS, and its level negatively correlated with Clara cell 10-kDa protein (CC10), another mucosa-associated peptide with known anti-inflammatory and immunomodulatory functions [11].

The role of TFFs has been most thoroughly studied in the GI tract, where increased TFF expression was found in the areas of chronic GI ulceration and underlying inflammation [12,13]. Cells found in those areas, called the ulcer-associated cell lineage, form a glandular structure that delivers its secretion products into the lumen via excretory ducts, including epidermal growth factor (EGF) and all three TFFs. EGF regulates transcription of all *TFF* genes trough a pathway mediated by epidermal growth factor receptor (EGF-R) and the Ras/mitogen-activated protein kinase (MEK)/mitogen-activated protein kinase (MAPK) signaling pathway [5]. To add to the complexity, there is evidence in vitro that TFF3 can initiate EGF-R mediated activation of MAPK and PI3K/Akt signaling pathways [14,15]. Although the role of TFFs in mucosal healing, especially in initiation of cell migration, is indisputable, the precise underlying molecular mechanisms and TFF receptors remain elusive. In addition, there is evidence for TFFs involvement in the innate and adaptive immune responses to microbes [16,17]. In the respiratory mucosa, TFF3 contributes to the antimicrobial defense by directly binding to microbes and promoting innate immune defense [18,19]. Taking all that into consideration and the fact that TFF expression is also abundant in the upper respiratory tract, it can be hypothesized that they could act in a similar fashion in the upper respiratory tract by promoting maintenance of the sinonasal epithelial barrier integrity, protecting against inflammation and nasal polyps formation [5,20]. Contrary to their beneficial physiological effects, there is emerging evidence that TFF peptides can also act as drivers of human cancer by acting as oncogenes and tumor suppressors, and thus they could potentially have an adverse role in dysregulation of sinonasal epithelia leading to the growth of benign tumors such as nasal polyps found in CRSwNP patients [8,9,10,21].

Data on TFF expression in sinonasal mucosa and nasal polyp tissue from CRSwNP are scarce, thus, the aim of this study was to assess expression levels of *TFF1* and *TFF3* genes in patients with CRSwNP and a control group of patients undergoing septoplasty. To assess the effect of surgical and postoperative intranasal steroid treatment on target gene expression, additional middle nasal turbinate samples were collected six months after functional endoscopic sinus surgery (FESS). Additionally, subgroups of CRSwNP patients with and without allergy and bacterial colonization of ethmoid sinus were specified. Here, we demonstrate differential expression of *TFF1* and *TFF3* genes in CRSwNP patients and an impact of the bacterial colonization on their expression.

## 2. Results

### 2.1. Characteristics of Subjects Enrolled in the Study

This study included 54 patients, of which 29 underwent FESS because of CRSwNP refractory to conservative treatment, and the remaining 25 patients were subjected to septoplasty because of insufficient respiration. On average, CRSwNP patients were significantly older as compared with the control patients undergoing septoplasty (53.4 ± 9.55 years old vs. 35.4 ± 11.1 years old, *p* < 0.001, Table 1), however, the observed difference reflects the age when patients typically undergo surgical treatment of CRSwNP or nasal deviation at our department and worldwide [1,22,23,24]. To ensure that the age differences had no significant effect on the TFFs expression, we performed a series of additional analysis of *TFF1* and *TFF3* mRNA levels in different age subgroups by employing a specialized software for comparison of multiple conditions (REST-MCS available at http://rest.gene-quantification.info/). The results showed no significant difference among the subgroups suggesting that *TFF1* and *TFF3* expression levels in sinonasal mucosa are not affected by age (Table S1 and Figure S1).

The ratio of man and woman in both groups showed a slight male dominance, without a significant difference between the groups (Chi-square = 0.951). Although the majority of patients in both groups where nonsmokers, frequency of smokers was significantly higher in the control group as compared with the CRSwNP group (Chi-square = 0.035, Table 1). However, as in the case of age, additional analysis revealed no significant effect of smoking on TFF expression levels (Figure S2).

Only six (20.7%) patients from the CRSwNP group had a positive radioimmunosorbent test (RIST, total IgE) and radioallergosorbent test (RAST, specific IgE), and proven eosinophils in nasal smear, which together with history of specific clinical signs and symptoms confirmed the diagnosis of allergic rhinosinusitis (Table 1). Patients from the control group that were positive for allergy or asthma were excluded from the study. The quality of life was assessed using the 20-item Sino-Nasal Outcome Test (SNOT-20) questionnaire, and the results showed similarly reduced quality of life in both groups (*p* = 0.374, Table 1). Six months after the surgery, CRSwNP patients reported sustained improvement of the life quality based on the SNOT-20 questionnaire (data not shown). Based on the CT grading score and endoscopy score (Lund–Mackay and the Malm classification, respectively) the majority of the patients who enrolled to participate in this study showed a moderate to severe form of nasal polyposis (Table 1).

### 2.2. Target Genes Expression in Nasal Polyps and Nasal Mucosa of CRSwNP Patients

In the CRSwNP group of patients, target gene expression levels were determined from tissue samples of nasal polyps (NP), middle nasal turbinate (MNT-0), and bulla ethmoidalis (BE) taken at the time of FESS surgery, and in the control MNT samples collected six months after surgical and continuous intranasal steroid treatment (MNT-6). In the case of the control group, the target genes mRNA levels were determined from inferior nasal turbinate samples (INT) taken at the time of septoplasty.

**Figure 1:**
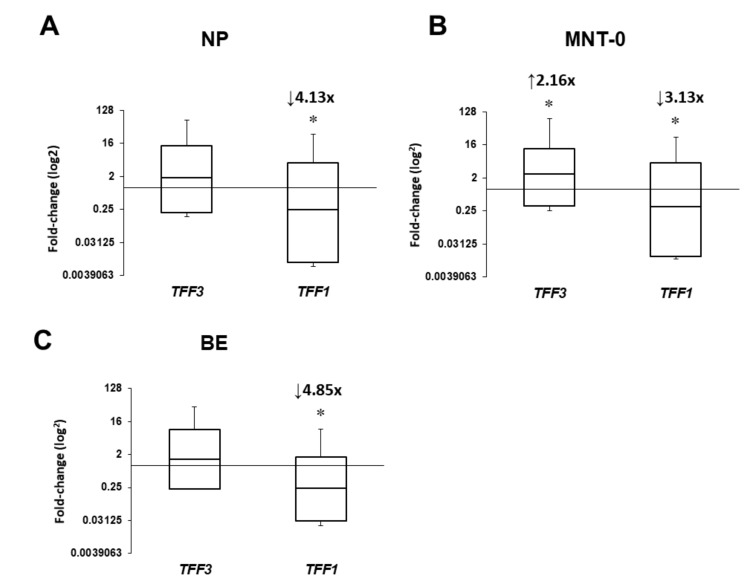
Expression of *TFF1* and *TFF3* in (**a**) nasal polyps (NP), (**b**) middle nasal turbinate (MNT-0), and (**c**) bulla ethmoidalis (BE) samples from CRSwNP patients relative to inferior nasal turbinate samples (INT) from the control group. The mRNA levels were compared using REST software (Qiagen), data are presented using whisker box (median, standard error, range), and * *p* < 0.05 was considered significant. ↑, up-regulated gene; ↓, down-regulated gene; CRSwNP, chronic rhinosinusitis with nasal polyps; NP, nasal polyp; MNT-0, middle nasal turbinate at the time of FESS; BE, bulla ethmoidalis.

**Figure 2:**
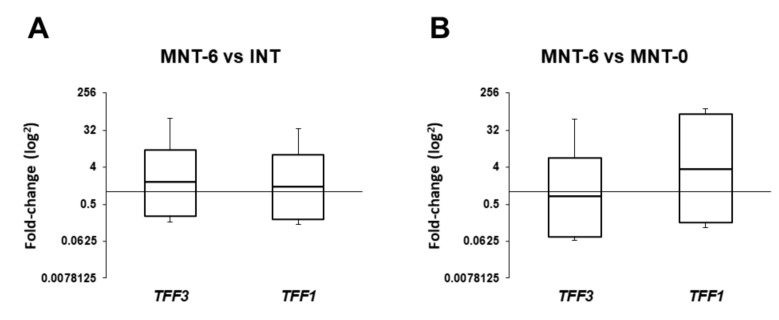
Expression of *TFF1* and *TFF3* in the middle nasal turbinate taken from CRSwNP patients six month following FESS and intranasal steroid therapy (MNT-6) relative to (**a**) inferior nasal turbinate samples (INT) from control group of patients and (**b**) middle nasal turbinate taken from CRSwNP patients at the time of FESS (MNT-0). The mRNA levels were compared using REST software (Qiagen) and data are presented using whisker box (median, standard error, range), *p* < 0.05 was considered significant. FESS, functional endoscopic sinus surgery; CRSwNP, chronic rhinosinusitis with nasal polyps; MNT-0, middle nasal turbinate of CRSwNP patients at the time of FESS; MNT-6, control samples of middle nasal turbinate collected six months after FESS from CRSwNP patients; and INT, inferior nasal turbinate from control group of patients.

*TFF1* and *TFF3* gene expression was detectable in all collected tissue samples from both CRSwNP and control patients. The mRNA levels of *TFF3* gene in the MNT-0 were significantly increased by approximately two-fold (*p* = 0.032, Figure 1B) as compared with the INT samples of the control group. Conversely, the *TFF1* gene was significantly downregulated in all tissue samples from CRSwNP patients taken at the time of FESS surgery compared to the control INT samples, including NP (4.13-fold, *p* = 0.0001, Figure 1A), MNT-0 (3.13-fold, *p* = 0.036, Figure 1B) and BE (4.85-fold, *p* = 0.017, Figure 1C). Six months after FESS and following a six-month local intranasal corticosteroid therapy, the mRNA levels of *TFF1* and *TFF3* genes in the MNT-6 samples were comparable to the values determined in the INT specimen of the control group (Figure 2A). This was further confirmed by additional comparison of MNT-0 and MNT-6 samples that showed opposite *TFF1* and *TFF3* gene regulation, i.e., the *TFF1* mRNA levels were rising by 1.44-fold (*p* = 0.417), while *TFF3* levels dropped by approximately 3.5-fold relative to MNT-0 (*p =* 0.066, Figure 2B).

### 2.3. Effects of Bacterial Colonization and Allergy on the Target Genes Expression in Nasal Polyps and Nasal Mucosa of CRSwNP Patients

In this study, 79.3% of CRSwNP patients had positive sinus swabs (Table 1), of which 51.7% had isolated pathogenic bacteria (*n* = 15) and 27.6% had diagnosed colonization by nonpathogenic bacteria *S. epidermidis* (*n* = 8, Table 2). Among the pathogenic bacteria isolated strains were *Staphylococcus aureus, Escherichia coli, Group B Streptococcus haemolyticus, Morganella morganii, Enterobacter spp, Serratia marascenses, Proteus mirabilis, Enterobacter freundii, and Klebsiella oxytoca. TFF1* and *TFF3* expression levels in sinonasal mucosa and nasal polyps were compared between the patients with positive sinus swabs and the group of patients whose swabs were sterile. In the subgroup of patients with isolated pathogenic bacteria we did not find any significant difference. however, patients with isolated normal flora presented with a significant seven-fold upregulation of the *TFF3* gene in MNT-0 and NP samples (*p* = 0.004 and *p* = 0.034, respectively, Table 2), as compared with the same tissue specimen from patients with sterile swab.

Allergy had no significant effect on the target gene expression in our study (Figure S3).

### 2.4. TFF1 and TFF3 Proteins Expression in Nasal Polyps and Nasal Mucosa of of CRSwNP Patients

Morphometric analysis of TFF1 and TFF3 protein expression was assessed by IHC staining of NP and MNT-0 specimen from CRSwNP patients, and INT tissue samples from control subjects. In general, TFF1 and TFF3 immunostaining was detected in pseudostratified columnar ciliated epithelium with goblet cells (respiratory epithelium) of all tissue samples, with slight differences of signal intensity across the samples. In some tissue specimens, signal was also present in the submucosal glands or the lymphoid cells infiltrating connective tissue.

**Figure 3:**
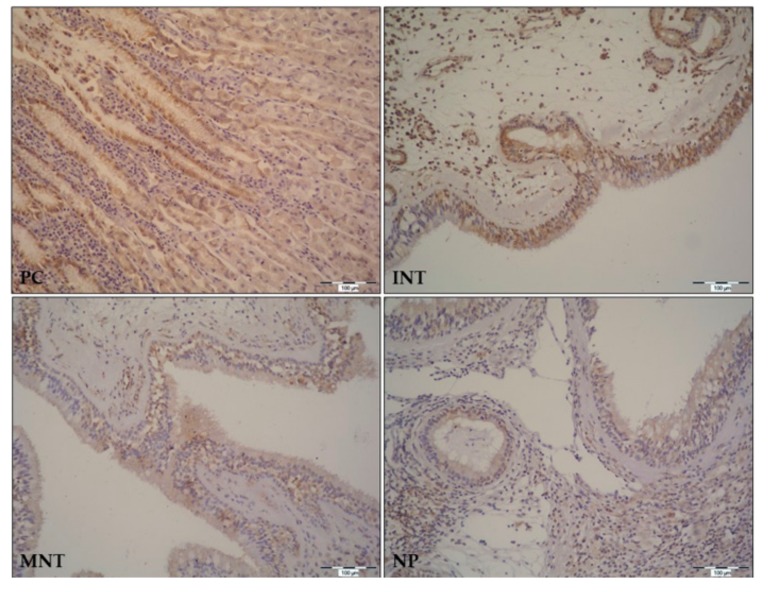
Localizations of TFF1 peptides in mucosa and submucosa of middle nasal turbinate (MNT) and nasal polyps (NP) from CRSwNP patients, and inferior nasal turbinate (INT) tissue from a control group of patients undergoing septoplasty. Gastric tissue was used as a positive control (PC). Formalin-fixed paraffin embedded tissue section were stained for TFF1 by immunohistochemistry using specific monoclonal anti-human TFF1 antibody and HRP/DAB detection dystem, and counterstained with hematoxylin. The positive signal for TFF1 protein is indicated by brown color and all figures are presented at 200× magnification. CRSwNP, chronic rhinosinusitis with nasal polyps. The representative IHC slides of MNT and NP are from patients with isolated *S. Epidermidis*.

In the INT tissue samples from the control group, TFF1 immunostaining was present in respiratory epithelium, submucosal glands, and lymphoid cells infiltrating underlying connective tissue (Figure 3). In the MNT-0 and NP tissue samples, TFF1 signal was detected in respiratory epithelium and submucosal lymphoid cells (Figure 3). Consistent with the gene expression results, TFF1 protein immunostaining was stronger in INT tissue samples as compared witht the MNT and NP tissue samples obtained from CRSwNP patients.

**Figure 4:**
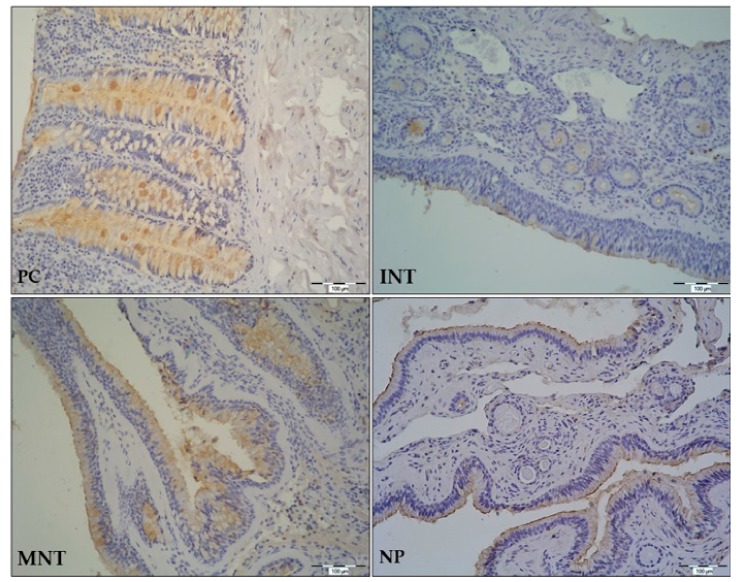
Localizations of TFF3 peptides in mucosa and submucosa of middle nasal turbinate (MNT) and nasal polyps (NP) from CRSwNP patients, and inferior nasal turbinate (INT) tissue from a control group of patients undergoing septoplasty. Colon tissue was used as a positive control (PC). Formalin-fixed paraffin embedded tissue section were stained for TFF3 by immunohistochemistry using specific polyclonal anti-human TFF3 antibody and HRP/DAB detection system, and counterstained with hematoxylin. The positive signal for TFF3 protein is indicated by brown color and all figures are presented at 200× magnification. CRSwNP, chronic rhinosinusitis with nasal polyps. The representative IHC slides of MNT and NP are from patients with isolated *S. Epidermidis*.

In the case of TFF3 immunostaining, specific signal was detected in the stratified squamous epithelium and the submucosal glands of INT from control subjects. In the MNT and NP tissue samples from CRSwNP patients signal was present only in the respiratory epithelium (Figure 4). When the TFF3 signal was compared among different tissue specimens, we found consistently stronger TFF3 signal in MNT tissue samples, as compared with INT and NP tissue samples (Table S2).

## 3. Discussion

In this study, we have investigated (a) expression levels of *TFF1* and *TFF3,* (b) tissue localization of the corresponding TFF peptides, and (c) the effects of bacterial colonization or infection and the presence of allergy on the target gene expression in sinonasal tissue from CRSwNP patients. Here, we present differential expression of target genes and significant effect of bacterial colonization by *S. epidermidis,* but not the presence of type 2 inflammation, on their mRNA levels. Briefly, compared to control samples, *TFF1* mRNA levels were significantly reduced in all specimens by approximately three- to five-fold, while *TFF3* was increased in MNT-0. Interestingly, six months following surgery and intranasal steroid therapy, their levels were reversed to control values. To the best of our knowledge, this is the first study with a representative number of CRSwNP patients and non-diseased controls exploring expression levels of *TFF1* and *TFF3* genes and peptides together with multiple clinical parameters to get the best correlation between TFF expression and its role in CRSwNP development.

In our study, we were able to detect mRNA levels of *TFF1* and *TFF3* genes and proteins in all samples from both CRSwNP (nasal polyps, bulla ethmoidalis, and middle nasal turbinate) and control patients (inferior nasal turbinate). These findings are in line with the results of several previous studies reporting substantial *TFF1* and *TFF3* expression in sinonasal mucosa [9,10]. The study by dos Santos Silva et al., in 1999, was the first to confirm the presence of *TFF1* and *TFF3* genes and peptides in the upper and lower respiratory tract of humans. In a multicenter study, they utilized tissue samples and sputum mainly from cystic fibrosis patients, several lung cancer patients, and only two samples of nasal polyp tissue from patients with chronic sinusitis undergoing polypectomy. As a control, normal bronchial tissue was collected from the upper respiratory tract of two patients undergoing lobectomy for bronchial carcinoma. They reported expression of *TFF1* and *TFF3* in material from all patients [9].

In our study, we have found a significant downregulation of *TFF1* gene in all tissue samples. This is in contrast to a study reported by Ping Li et al. who failed to find any significant change in the expression of *TFF1* and *TFF3* genes and peptides in a group of CRSwNP patients, while they reported significantly increased expression of both TFFs in CRSsNP patients as compared with a group of non-diseased subjects [10]. Their results were based on ethmoid sinus mucosa only, which might be partially the reason for the observed discrepancies. A recent study reported increased *TFF1* gene and protein expression in patients suffering from either CRS or nasal polyposis without CRS, but did not explore *TFF1* expression in the CRSwNP patients [11]. During CRSwNP one would expect increased TFF1 secretion in response to inflammatory injury of the respiratory mucosa, however, our results did not support this hypothesis, and the possible explanation could be an inverse adaptation to excessive nasal mucosa outgrowth.

The results of our study revealed significantly increased *TFF3* mRNA levels in the nasal mucosa of CRSwNP patients. Among them, a subgroup of CRSwNP patients with *S. epidermidis* isolated from sinus swabs presented with more than seven-fold higher expression levels of *TFF3* in MNT and NP as compared to patients with sterile swabs. These findings match the results published in the aforementioned paper by dos Santos Silva et al. who confirmed the presence of TFFs in normal and inflamed mucosa. Additionally, they reported direct binding of TFF3 onto the bacteria *S. aureus* and *P. aeruginosa* in sputum samples of patients with cystic fibrosis, which may contribute to the defense mechanisms of the innate immune system [9]. Nevertheless, the role of *S. epidermidis* as an etiological factor of CRS with or without nasal polyposis is controversial. Generally, *S. epidermidis* is considered to be an important commensal organism of the human skin and mucous membranes, with an emerging role in controlling expansion of pathogenic microorganisms, such as *S. aureus* and *Group A Streptococcus*. Conversely, *S. epidermidis* can cause opportunistic infections, which include particularly biofilm-associated infections on indwelling medical devices [25]. It has been proposed that during *S. epidermidis* colonization of the adult skin, factors produced by *S. epidermidis* activate pattern recognition receptors mediated signaling cascades that result in the production of cytokines and the antimicrobial peptides such as β-defensins. Similarly, it has been shown, in the gut, that secretion of IL-6 in response to commensal bacteria protects the intestinal epithelium from injury, possibly by regulating intestinal TFF secretion [18,19,26,27]. Considering that bacterial infection and formation of biofilms is one of the predisposing factors in the cascade of events that leads to chronic rhinosinusitis, we can assume that TFFs, and particularly TFF3, have an important role in defending the epithelial surface from bacterial colonization. It is worth noting that six months after surgery mRNA levels of *TFF1* and *TFF3* genes in collected samples were comparable to the results of the control group. Considering that management and surgery outcomes of patients with CRSwNP are sometimes limited [28,29] and affect the quality of life of those patients we see a potential for utilization of TFF expression as a biomarker for assessing surgery outcomes. 

Results of a study by Miyahara N et al. revealed significantly increased expression levels of TFF1 and TFF3 in the nasal mucosa of individuals with proven allergy as compared with subjects without allergy. Furthermore, they reported an association of *TFF1* and *TFF3* expression with *MUC5AC* and *MUC5B* mRNA levels, leading to a conclusion that upregulation of TFF1 and TFF3 and MUC5B may play an important role in the development of type 2 inflammation in nasal mucosa [30]. These results are in contrast to our finding that the *TFF1* and *TFF3* genes were not differentially expressed in subjects with evident allergic rhinosinusitis. A possible explanation for the observed discrepancies could be the different pools of patients used, i.e., our results were gained from patients suffering from CRSwNP, whereas Miyahara N and colleagues formed their cohort from patients undergoing turbinectomy.

In the GI tract, TFF peptides are expressed in surface epithelial cells, surface mucous cells, and goblet cells. A similar pattern of expression is found in the upper and lower respiratory tract mucosa where expression of TFF peptides can be found in epithelial goblet cells, ciliated cells, on cilia, and in some submucosal cells [6]. In our study, TFF1 and TFF3 were identified in the ciliated epithelium with goblet cells, submucosal glands, and lymphoid cells infiltrating underlying connective tissue. We would like to emphasize that our study is the first to assess and analyze the expression pattern of *TFF1* and *TFF3* genes and peptides in a representative number of nasal polyp tissues collected from CRSwNP patients. Immunostaining of both peptides was evident in nasal polyps, and the expression pattern was similar to the one found in nasal mucosa. Considering that expression of TFFs is still continued in NP, we can assume that tissue remodeling does not end with polyp formation but continues and probably adds to polyp growth. Our study provides additional evidence for the important role of TFFs in tissue remodeling of the respiratory system, which, as proposed by Hoffman W. [31], makes them promising targets for pharmaceutical interventions in acute and chronic airway diseases.

CRSwNP is a chronic inflammatory condition with known dysregulation of IL-6 pathway [32]. This could be a possible link to the observed differences in *TFFs* expression in our study, since some previous studies demonstrated a tight regulation of TFF3 secretion through the IL-6-STAT3 pathway [33,34]. In a study by Jiang GX et al., exposure of human biliary epithelial cells (BECs) to recombinant IL-6 resulted in STAT3 phosphorylation and increased expression of *TFF3* gene and protein, and this effect could be abolished by RNA interference [33]. Reciprocal negative regulation between the intracellular IL-6/gp130 signaling pathways, STAT3, and mitogen-activated protein kinase (MAPK) is well known from other immune response studies, and further confirmed in a study by Nozaki I. and colleagues where they employed IL-6+/+ and IL-6−/− mice subjected to chronic bile duct ligation and showed that BEC TFF3 expression is dependent primarily on STAT3 signaling [34]. They concluded that use of recombinant IL-6 or TFF3 peptides had a therapeutic role in preventing biliary strictures in liver allografts. These findings suggest that increased IL-6 levels could, in addition to a known pro-inflammatory effect, also have a pivotal role in the process of tissue resuscitation during chronic inflammation. 

In conclusion, our study showed that TFF1 and TFF3 peptides are continuously expressed in NP tissue in the same fashion as in the upper and lower respiratory tract and the GI tract [35,36]. We have detected differential expression of *TFF1* and *TFF3* genes and peptides in sinonasal mucosa of patients with CRSwNP, where they could play an important role in mucosal defense from bacterial infection and the development of chronic rhinosinusitis. Considering that *TFF* expression levels after surgery and continuous intranasal steroid therapy were comparable to control values, we suggest the potential use of TFF expression as an indicator for long-term follow-up of treatment outcomes in patient with CRSwNP.

## 4. Materials and Methods

### 4.1. Study Design

This is a prospective study exploring patients with CRSwNP subjected to functional endoscopic sinus surgery (FESS) during a one-year period (from the 1 October 2012 to 30 September 2013) at the Department of Otorhinolaryngology and Head and Neck Surgery of the University Hospital Osijek (Croatia). An additional group of patients undergoing nasal septoplasty because of the correction of anatomic variations, such as deviated nasal septum or hypertrophic lower turbinate, was employed to form the control group. The study was approved by the Ethics committee of the Faculty of Medicine at the University of Osijek (Croatia) and the Ethics committee of the University Hospital Osijek (no. 25-1:11709-2/2010 issued on 13 December 2010) assuring that the study was planned and carried out in accordance with the principles laid down in the Declaration of Helsinki. All patients provided signed informed consent.

### 4.2. Subjects

An illustration of study groups and study protocol is given in Figure S4. Patients (29 CRSwNP and 25 control group) examined at the Department of Otorhinolaryngology and Head and Neck Surgery of the University Hospital Osijek (Croatia) were recruited to participate in this study. Initially, they were examined by the anterior rhinoscopy and endoscopy, and underwent all standard laboratory diagnostics, including blood tests, nasal smear for eosinophils and microbiological assessment, serum total and specific IgE. Patients were treated according to the recommendations given in the European position paper on rhinosinusitis and nasal polyps [1]. Accordingly, conservative therapy of CRSwNP preceded surgical treatment. During a six-month period, patients were advised to use intranasal steroid therapy, and in some cases additionally instructed to use antibiotics. Furthermore, all patients were uniformly using local (mometasone furoate) and systemic steroids (15, 10, 5 mg oral prednisone) during a period of 3 weeks before surgical treatment. In the case of an unsatisfactory result, the patient underwent FESS and, following surgery, continued to take local intranasal steroids for a minimum of six months. The indication for performing FESS on CRSwNP patients was unsatisfactory response to medicamentous treatment during a six-month period with persistent symptoms and reduced quality of life, whereas the indication for septoplasty was insufficient nasal respiration. In the case of CRSwNP patients, samples of nasal polyps (NP), middle nasal turbinate (MNT-0), and bulla ethmoidalis (BE) during surgery, and a control MNT sample 6 months postoperatively (MNT-6) were collected. Mucosal punctuates, from lower turbinate (inferior nasal turbinate, INT) acquired from control patients, were used as control specimens.

The CRSwNP and control patients were operated on by FESS using Wigand technique and septoplasty using Cottle technique, respectively (Karl Storz and Wolf operating equipment). Tissue specimens for the qPCR and immunohistochemical analysis were collected during the surgery by the same investigator and processed in the same fashion. Tissue biopsies were taken from the corresponding region in the MNT, INT, or anterior ethmoid sinuses using a standard-sized Blakesley forceps (size 3, cup size 5 × 15 mm; Karl Storz, Tuttlingen, Germany). An additional control sample was taken from CRSwNP patients 6 months after the surgery, under locally applied anaesthesia. Exclusion criteria in both groups were the following: patients under the age of 18, systemic disease involving the nose (Wegener’s granulomatosis, cystic fibrosis, Kartageners syndrome, sarcoidosis), primary ciliary dyskinesia, pregnancy, or ongoing treatment for cancer. In order to assess the influence of allergy and bacterial colonization on the target genes and proteins expression in CRSwNP patients, additional exclusion criteria for the control group patients were proven allergy or bacterial or fungal colonization of the nasal cavity. Information regarding patients’ gender, age, smoking, history of asthma, SNOT 20 analysis, CT Lund–Mackey and Malm classification total nasal endoscopy score (both only for CRSwNP patients), and the course of the surgical procedure and the postoperative period were collected.

### 4.3. Quantitative Real-Time Polymerase Chain Reaction

Tissue samples from different anatomical locations (as described in a previous section) were collected during the surgery at the Department of Otorhinolaryngology and Head and Neck Surgery of the University Hospital Osijek. Tissue was preserved in RNA later solution (Qiagen, Hilden, Germany) to prevent RNA degradation for 2 to 4 h at RT and overnight at +4 °C and stored at −80 °C until processed for qPCR. Total RNA was isolated using a RNeasy Mini kit (Qiagen), according to the manufacturer’s instructions. 1.5 µg of RNA was transcribed into cDNA using a High-Capacity cDNA Reverse Transcription Kit (Applied Biosystems, Dreieich, Germany). Quantitative reverse transcription polymerase chain reaction (qRT-PCR) was performed using SYBR Green I (Invitrogen, Waltham, MA, USA) detection chemistry and specific primers (Table 3) on a Real-time PCR System 7300 (Applied Biosystems, SAD, Dreieich, Germany). Primer pairs were designed to give only one band (confirmed by gel electrophoresis and melting curve analysis) and conditions of reactions were optimized so that efficiency of PCR reaction was 95% to 100% Target genes were *TFF1* and *TFF3*, and the house keeping genes were *HPRT*, *18S*, *GUS*, *YWHAZ*, and *RPL13A*. On the basis of the GeNorm analysis, all data were corrected to *HPRT* and *YWHAZ* as the most stable reference genes. The cycling conditions comprised 3 min polymerase activation at 95 °C and 40 cycles including 95 °C for 1 min, annealing temperature specific for each primer pair (Table 3) for 30 s, and elongation at 72 °C for 30 s. A single product amplification was confirmed by melting curve analysis. Gene expression was analyzed by REST-MCS software (version 2, http://rest.gene-quantification.info) using ΔΔ*C*t method [37,38] and normalized to stable housekeeping genes, β-actin and β2-microglobulin. Changes were represented as log_2_ (fold change).

### 4.4. Immunohistochemistry

Tissue samples were taken intraoperatively (as described in previous sections), fixed in 10% formaldehyde for 72 h, and embedded in paraffin. Prior to IHC staining, paraffin blocks were cut into 6 µm thick sections and transferred to the polysine adhesive slides (Thermo Fisher, Waltham, MA, USA). Slides were dried, deparaffinized (using various dilutions of absolute ethanol) and rehydrated in phosphate-buffered saline (PBS; pH 7.4). Epitope retrieval was performed by heating in the microwave oven for 3 min, using 0.01 M citric acid (pH 6). Following, endogenous peroxidase blocking step and nonspecific protein blocking step were performed using 3% H_2_O_2_ for 15 min and SuperBlock^®^ (Thermo Scientific, Rockford, IL, USA) for 60 min, respectively. Affinity purified primary polyclonal rabbit anti-human and anti-mouse TFF3 antibody (in house Ab, specificity confirmed on Tff3 deficient mice) and monoclonal mouse anti-human TFF1 antibody (clone p2802, kindly provided by C.L. Tomasseto) were applied to separate slides and incubated at 4 °C overnight. The antibody specificities were confirmed by immunostaining of the gastric mucosa and the colon mucosa, in the case of anti-TFF1 and the anti-TFF3 antibody, respectively. Antibodies were diluted in 1x PBS at a dilution rate of 1:50 for anti-TFF1, and 1:10,000 for anti-TFF3. Slides with omitted primary antibodies were used as negative controls for each patient. Secondary antibodies were applied on the second day, following 4 (5 min) washing steps with PBS + 0.05% Tween (Sigma-Aldrich, St. Louis, MO, USA). In the case of primary anti-TFF1, the biotin goat anti-mouse antibody (BD Bioscience, US, 2h at RT), and in the case of primary anti-TFF3, the biotinylated goat anti-rabbit secondary antibody (Dako, Glostrup, Denmark; 30 min at RT) were used. After 4 × 5 min washes in PBS + 0.05% Tween, Streptavidin-HRP (Dako, Glostrup, Denmark) was applied to the slides (30 min at RT). Next, slides were washed, exposed to DAB (3,3’-diaminobenzidine) (Sigma-Aldrich, St. Louis, MO, USA) for 5 min, and immediately rinsed in PBS + 0.05% Tween. Finally, the slides were counterstained with hematoxylin, dehydrated, and mounted with Canada balsam. Slides were than analyzed and photographed at 100× and 200× magnification (Olympus^®^ BX50 microscope, Olympus^®^ C-5050 digital camera and QuickPHOTO PRO imaging software; Promicra s.r.o., Prague, Czech Republic). MS Power Point software (Microsoft Office Professional Plus 2016, Microsoft Corporation, Redmond, WA, USA) was used for image assembly.

### 4.5. Nasal Smear Foe Eosinophils

Nasal smear was performed by swabbing both nostrils, smearing it on the glass slide, and drying. Following, the slides were sent to the Laboratory for Citology at the Clinical Department for Citology of the University Hospital Osijek (Croatia). Glass slides were stained with standard eosinophilic buffer and analyzed under a light microscope (Olympus BX50 Light Microscope, Prague, Czech Republic).

### 4.6. Acquisition of Nasal and Sinus Swabs

In our study we have performed nasal and sinus swabs for bacterial cultivation and identification. In the case of CRSwNP patients, we took sinus swabs of the ethmoid sinus intraoperatively, whereas in the control group of healthy subjects we performed nasal swab preoperatively. Endoscopically guided sinus swabs were always taken from the anterior ethmoid sinus. We used flocked swabs (Copan Italia S.p.A., Brescia, Italy) to maximize bacterial yield. To avoid inadvertent contamination, any swabs that could have come into contact with the nasal vestibule during sampling were discarded. Among the patients who participated in our study, there was none with purulent secretion. Samples were analyzed in a routine microbiological laboratory at the Institute of Publich Healh Osijek-baranja County (Croatia).

## Figures and Tables

**Table 1:** Characteristics of the patients enrolled in the study.

	CRSwNP	Control
*N*	29	25
Age * (years old)	53.4 ± 9.55 (26–69)	35.4 ± 11.1 (21–60)
Gender (M/F)	16/13	14/11
Smokers	4 (13.8%)	10 (40.0%)
RIST/RAST positive	6 (20.7%)	0
AERD	3 (10.3%)	0
Sinus swab positive	23 (79.3%)	N/A
Sinus swab-sterile	6 (20.7%)	N/A
Nasal swab-sterile	N/A	25 (100%)
SNOT-20 *	41.6 ± 25.9 (6–86)	35.9 ± 19.8 (13–77)
CT grading score *	12.0 ± 5.19 (2–24)	-
Total endoscopy score *	4.10 ± 2.16 (0–6)	-

N, number of patients; CRSwNP, chronic rhinosinusitis with nasal polyposis; RIST, radioimmunosorbent test (total IgE); RAST, radioallergosorbent test (specific IgE); AERD, aspirin-exacerbated respiratory disease, also known as Samter’s Triad; SNOT-20, 20-item sino-nasal outcome test. CT score was obtained using Lund–Mackay classification and endoscopy score was determined according to the Malm classification. * Data are presented as mean ± SD (range) and N/A, not applicable.

**Table 2:** Effects of bacterial colonization of the ethmoidal sinus on the *TFF1* and *TFF3* gene expression in collected specimens.

Sample	Middle Nasal Turbinate	Bulla Ethmoidalis	Nasal Polyp
Pathogenic Bacteria (*n* = 15)	n.a.	n.a.	n.a.
Normal Flora (*n* = 8)	↑↑*TFF3* (7.16x; *p = 0.004*)	-	↑↑*TFF3* (7.77x; *p = 0.034*)

Data were compared to the same mucosa sample from patients whose sinus swab remained sterile (*n* = 6) using REST 2009 software; *p* < 0.05 was considered significant; ↑, up-regulated gene; ↓, down-regulated gene and n.a., not affected.

**Table 3:** Oligonucleotide primers used for Q-PCR analysis.

Gene		Sequence 5’–3’	Optimized PCRcondition (Annealing Temp/ MgCl_2_)
*TFF1*	For	TTTGGAGCAGAGAGGAGGCAATG	57 °C/3.5 mM
	Rev	ACCACAATTCTGTCTTTCACGGGG	
*TFF3*	For	CTTGCTGTCCTCCAGCTCT	57 °C/3.5 mM
	Rev	CCGGTTGTTGCACTCCTT	
	Rev	ACGAGTCAGAGCTTTGGCTAGGAA	
*HPRT1*	For	GCTTTCCTTGGTCAGGCAGTAT	58 °C /3.5 mM
	Rev	GGCTTATATCCAACACTTCGTGGG	
*18S rRNA*	For	GTAACCCGTTGAACCCCATT	59 °C/3 mM
	Rev	CCATCCAATCGGTAGTAGCG	
*GUS*	For	TCACCAGGATCCACCTCTGATGTT	59 °C/3.5 mM
	Rev	TTGGTTGTCTCTGCCGAGTGAAGA	
*YWHAZ*	For	CCCACAGACTATTTCCCTCATC	59 °C/3 mM
	Rev	GACAGACCATTCAGGATAGGTAGG	
*RPL13A*	For	CCTGGAGGAGAAGAGGAAAGAGA	59 °C/3 mM
	Rev	TTGAGGACCTCTGTGTATTTGTCAA	

TFF1, trefoil factor family protein 1; TFF3, trefoil factor family protein 3; HPRT1, hypoxanthine phosphoribosyltransferase 1; 18S, 18S ribosomal RNA; GUS, beta-glucuronidase; YWHAZ, tyrosine 3-monooxygenase/tryptophan 5-monooxygenase activation protein zeta; RPL13A, ribosomal protein L13a.

## Supplementary Materials

Supplementary materials can be found at https://www.mdpi.com/1422-0067/20/21/5461/s1.

## Abbreviations

AERD	Aspirin-exacerbated respiratory disease
BE	Bulla ethmoidalis
CRS	Chronic rhinosinusitis
CRSwNP	Chronic rhinosinusitis with nasal polyposis
CRSsNP	Chronic rhinosinusitis without nasal polyposis
EGF	Epidermal growth factor
FESS	Functional endoscopic sinus surgery
18S	18S ribosomal RNA
GI	Gastrointestinal
GUS	Beta-glucuronidase
HPRT1	Hypoxanthine phosphoribosyltransferase 1
INT	Inferior nasal turbinate
MNT	Middle nasal turbinate
NP	Nasal polyps
RAST	Radioallergosorbent test
RIST	Radioimmunosorbent Test
RPL13A	Ribosomal Protein L13a
TFF1	Trefoil family factor protein 1
TFF2	Trefoil factor family protein 2
TFF3	Trefoil factor family protein 3
YWHAZ	Tyrosine 3-monooxygenase/tryptophan 5-monooxygenase activation protein zeta
