# Supplementary material for: Differential Expression of TFF1 and TFF3 in Patients Suffering from Chronic Rhinosinusitis with Nasal Polyposis
Source: Int J Mol Sci. 2019 Nov 1;20(21):5461. doi: 10.3390/ijms20215461 (PMC6862153; doi:10.3390/ijms20215461)
Supplement: Supplementary file 1 [file ijms-20-05461-s001.pdf]

Supplemental Table 1. Patients subgroups based on age at the time of enrollment into the study

	CRSwNP			Control	
	Group 1 (≤50 years)	Group 2 (51-55 years)	Group 3 (≥56)	Group 1 (≤40 years)	Group 2 (≥41)
**Mean**	42,29	52,13	62,08	28,31	49,75
**StDev**	8,22	0,83	4,44	4,38	5,52
**Count**	8	9	12	16	9
**Min**	27	51	57	21	41
**Max**	50	53	70	37	60

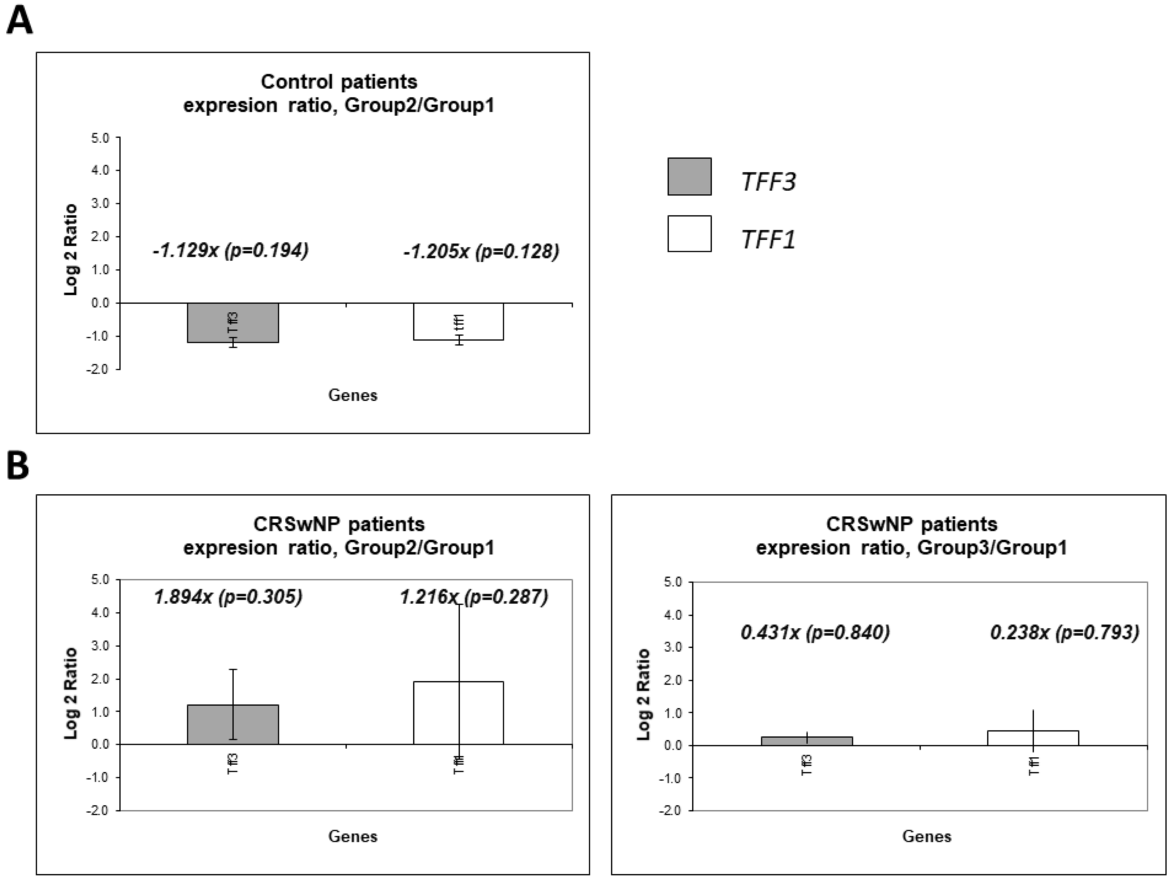

Supplemental Figure 1. Effect of age on the *TFF1* and *TFF3* expression

There was no difference in *TFF1* and *TFF3* expression among the age subgroups of control (Suppl. fig 1A) and CRSwNP (Suppl. fig. 1B) patients. Based on the age 3 subgroups of CRSwNP (Group 1 - 42.29±8.2, Group - 2 52.13±0.83, Group 3 - 62.08±4.44) and 2 subgroups of control patients (Group 1 - 28.31±4.38, Group 2 - 49.75±5.52) were identified. mRNA levels were compared using REST software (Qiagen), data are presented as Mean and Standard. Error, and p<0.05 was considered significant. CRSwNP – Chronic Rhinosinusitis with nasal polyps.

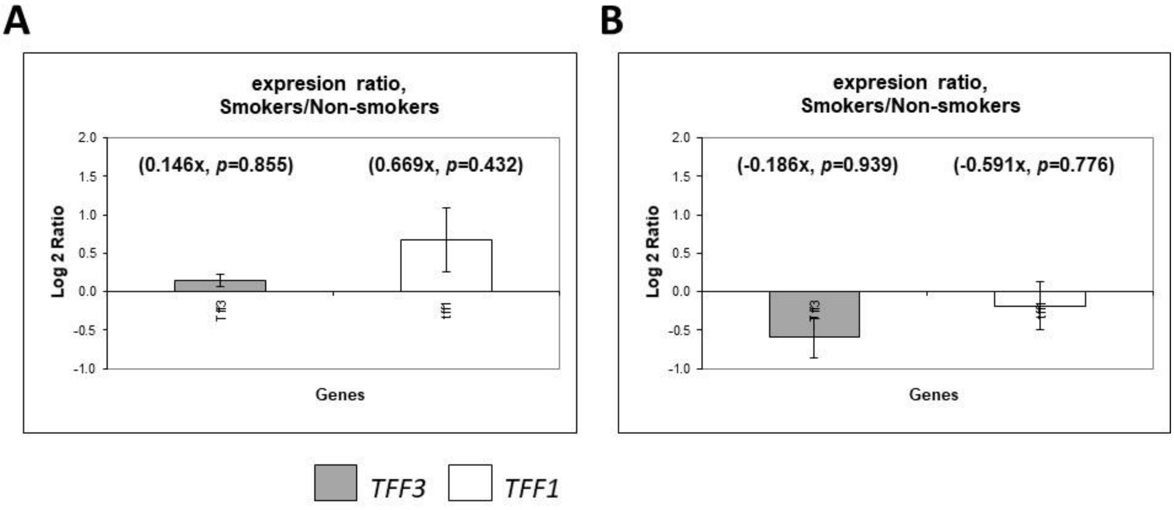

Supplemental Figure 2. The effect of smoking on *TFF1* and *TFF3* expression

The TFF1 and TFF3 expression levels were compared between smokers and non-smokers in control (Suppl. fig 2A) and CRSwNP (Suppl. fig. 2B) group of patients. The data are presented as fold-change in subgroup of smokers relatively to the subgroup non-smokers. mRNA levels were compared using REST software (Qiagen), data are presented as Mean and Standard. Error, and p<0.05 was considered significant.

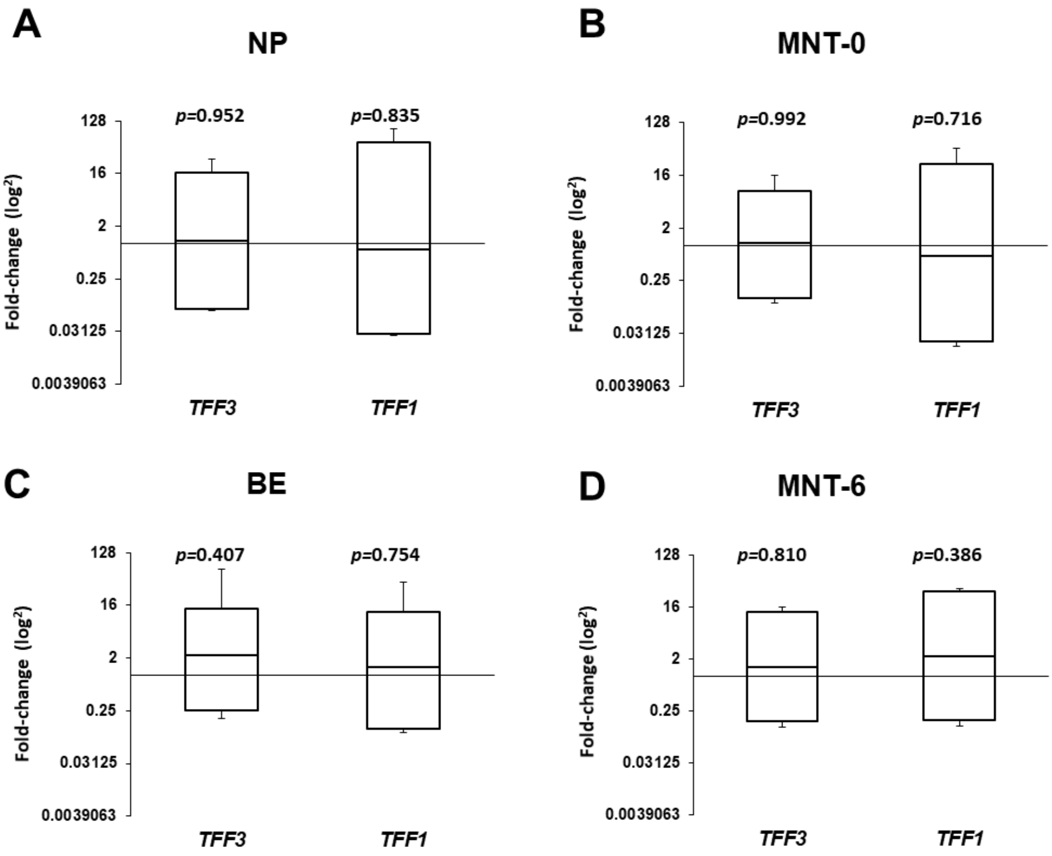

Supplemental Figure 3. The effect of allergy on *TFF1* and *TFF3* Expression in a) nasal polyps (NP), b) middle nasal turbinate (MNT-0), c) bulla ethmoidalis (BE) and d) control samples of middle nasal turbinate taken after 6 months (MNT-6) from CRSwNP patients. The data are presented as fold-change in subgroup of patients diagnosed with allergy relatively to the subgroup of patients without allergy. mRNA levels were compared using REST software (Qiagen), data are presented using

Whisker box (Median, Standard. Error, Range), and p<0.05 was considered significant. CRSwNP – Chronic Rhinosinusitis with nasal polyps. NP- nasal polyp, MNT-0 – middle nasal turbinate at the time of FESS, BE – bulla ethmoidalis, MNT-6 – control samples of middle nasal turbinate collected six months after FESS from CRSwNP patients.

Supplemental Table 2: Histomorphological analysis of TFF1 and TFF3 protein expression by Modified Quick Score (Q) method for immunohistochemistry protein expression analysis

#	TFF1			TFF3		
	MNT	NP	INT	MNT	NP	INT
**1**	4	3	5	6	4	2
**2.**	3	4	6	5	2	3
**3.**	4	4	5	7	3	5
**4.**	5	4	4	5	3	4
**5.**	4	3	5	6	4	4
**6.**	3	4	6	6	3	4
**7.**	3	5	6	6	4	2
**8.**	2	3	6	7	3	3
**9.**	4	4	5	6	4	3
**10.**	4	4	7	5	5	3
**Mean**	**3,60**	**3,80**	**5,50**	**5,90**	**3,50**	**3,30**
**StDev**	**0,84**	**0,63**	**0,85**	**0,74**	**0,85**	**0,95**
**Count**	**10,00**	**10,00**	**10,00**	**10,00**	**10,00**	**10,00**
**SEM**	**0,27**	**0,20**	**0,27**	**0,23**	**0,27**	**0,30**

Proportion score (A): 0 - No signal <1% - 0; 1 = 1%-10% of positive cells ; 2 - 11%-33%; 3 -34%-66%; 4 - 67 %-100%; **Intensity score (B):** 0 - No signal; 1- Weak signal; 2 - Moderately strong signal; 3 – Strong signal; Q = A + B, where Q - Quick Score total result; A = proportion score; B = intensity score; minimum Q score was 0 and maximum was 7.

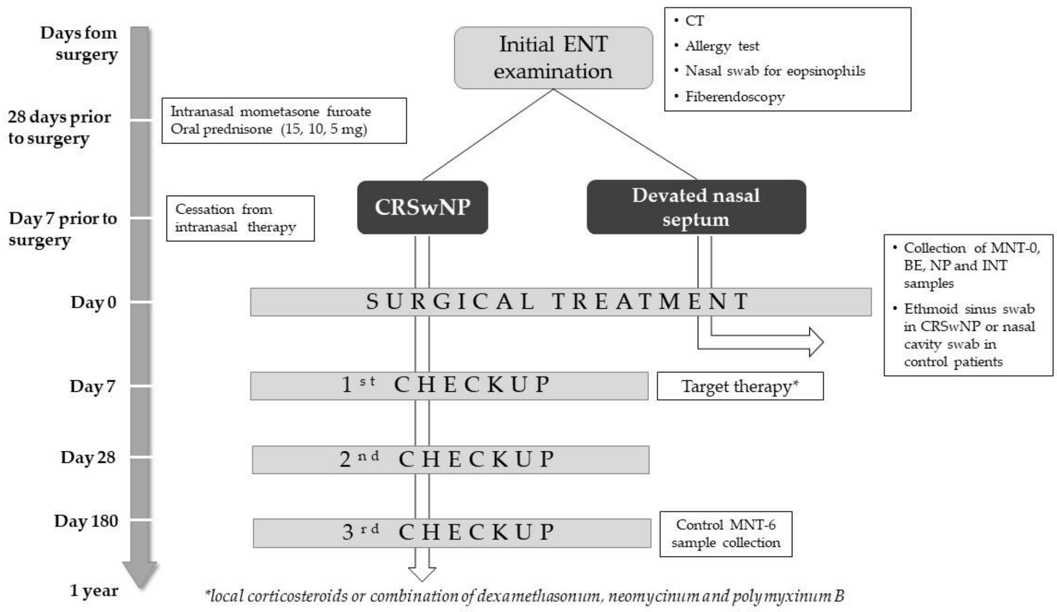

Supplemental Figure 4. Schematic representation of the study protocol. 54 patients (29 CRSwNP and 25 control group) examined at the Department of Otorhinolaryngology and Head and Neck Surgery University Hospital Osijek (Croatia) were recruited to participate in this study. Initially, they were **examined by the anterior rhinoscopy and endoscopy**, **and underwent all standard laboratory diagnostics, including blood tests, nasal smear for eosinophils and microbiological assessment, serum total and specific IgE**. CRSwNP patients were diagnosed and treated according to the European Position Paper on Rhinosinusitis and Nasal Polyp guidelines published in 2012. Accordingly, conservative therapy of CRSwNP preceded surgical treatment. During a 6-months period patients were advised to use intranasal steroid therapy, and in some cases additionally instructed to use antibiotics. Furthermore, all patients were uniformly using local (mometasone furoate) and systemic steroids (15, 10, 5 mg oral prednisone) during a period of 3 weeks before surgical treatment.. Indication for performing FESS on CRSwNP patients was unsatisfactory response to intranasal corticosteroid sprays and antibiotics during a six-month period with persistent symptoms and reduced quality of life, while indication for septoplasty was insufficient nasal respiration. Following FESS surgery CRSwNP patients continued to use local intranasal steroids for a minimum of six months **Tissue specimens for the qPCR and immunohistochemical analysis were harvested during the surgery by the same investigator and processed in the same fashion.** An additional **control sample was taken from CRSwNP patients 6 months after the surgery**. In the case of CRSwNP patients, samples of nasal polyps (NP), middle nasal turbinate (MNT-0) and Bulla ethmoidalis (BE) were collected during surgery, while a control MNT sample was taken 6 months postoperatively (MNT-6), under locally applied anesthesia. Mucosal punctuates of lower turbinate (inferior nasal turbinate, INT) acquired from control patients were used as control specimens.

FESS – Functional Endoscopic Sinus Surgery, **CRSwNP**– Chronic Rhinosinusitis with nasal polyps. **MNT-0** – middle nasal turbinate of CRSwNP patients at the time of FESS, **MNT-6** – control samples of middle nasal turbinate collected six months after FESS from CRSwNP patients; **INT** – inferior nasal turbinate from control group of patients.

I.D.:________________ **SINO-NASAL OUTCOME TEST (SNOT-20)** DATE:______________

Below you will find a list of symptoms and social/emotional consequences of your rhinosinusitis. We would like to know more about these problems and would appreciate your answering the following questions to the best of your ability. There are no right or wrong answers, and only you can provide us with this information. Please rate your problems as they have been over the past two weeks. Thank you for your participation. Do not hesitate to ask for assistance if necessary.

1. Considering how severe the problem is when you experience it and how frequently it happens, please rate each item below on how "bad" it is by circling the number that corresponds with how you feel using this scale: →	No problem	Very mild problem	Mild or slight problem	Moderate Problem	Severe Problem	Problem as bad as it can be	5 Most Important Items
**1. Need to blow nose**	0	1	2	3	4	5	○
**2. Sneezing**	0	1	2	3	4	5	○
**3. Runny nose**	0	1	2	3	4	5	○
**4. Cough**	0	1	2	3	4	5	○
**5. Post-nasal discharge**	0	1	2	3	4	5	○
**6. Thick nasal discharge**	0	1	2	3	4	5	○
**7. Ear fullness**	0	1	2	3	4	5	○
**8. Dizziness**	0	1	2	3	4	5	○
**9. Ear pain**	0	1	2	3	4	5	○
**10. Facial pain/pressure**	0	1	2	3	4	5	○
**11. Difficulty falling asleep**	0	1	2	3	4	5	○
**12. Wake up at night**	0	1	2	3	4	5	○
**13. Lack of a good night's sleep**	0	1	2	3	4	5	○
**14. Wake up tired**	0	1	2	3	4	5	○
**15. Fatigue**	0	1	2	3	4	5	○
**16. Reduced productivity**	0	1	2	3	4	5	○
**17. Reduced concentration**	0	1	2	3	4	5	○
**18. Frustrated/restless/irritable**	0	1	2	3	4	5	○
**19. Sad**	0	1	2	3	4	5	○
**20. Embarrassed**	0	1	2	3	4	5	○

2. Please mark the most important items affecting your health (maximum of 5 items)______________________ ↑

Copyright © 1996 by Jay F. Piccirillo, M.D., Washington University School of Medicine, St. Louis, Missouri
